# Autophagy: From Molecular Mechanisms to Disease Regulation and Therapeutic Strategies

**DOI:** 10.3390/cimb48030285

**Published:** 2026-03-07

**Authors:** Huijie Yang, Xinyu Li, Kaidie Wang, Yujiao Zou, Quanjuan Shi, Ya Yang, Qingyun Zhao, Wei Zou

**Affiliations:** School of Public Health, Kunming Medical University, Kunming 650500, China; huijieyang0601@outlook.com (H.Y.); xinyuliyx@163.com (X.L.); 19169333712@163.com (K.W.); z3431018056@outlook.com (Y.Z.); shiquanjuan0425@outlook.com (Q.S.); 18314262861@163.com (Y.Y.)

**Keywords:** autophagy, molecular mechanisms, microbiota–gut–brain axis, neurodegenerative diseases, metabolic disorders, therapeutic strategies

## Abstract

Autophagy is increasingly recognized as a context-dependent regulatory process that links cellular quality control with systemic metabolic and neurological homeostasis. However, how distinct autophagy pathways contribute to disease progression, and how they are dynamically modulated by host–microbiota interactions, remain incompletely understood. In this review, we synthesize recent advances in the molecular regulation of macroautophagy, microautophagy, and chaperone-mediated autophagy (CMA), with a particular emphasis on selective autophagy and its disease-specific functions. We examine emerging evidence implicating autophagy as a bidirectional modulator in neurodegenerative and metabolic disorders, highlighting conditions under which autophagy exerts protective versus maladaptive effects. Importantly, we integrate recent findings on the microbiota–gut–brain axis to illustrate how microbial signals reshape autophagic responses and influence disease susceptibility and progression. Finally, we summarize current progress and limitations in autophagy-targeted therapeutic strategies, including nanomedicine-based delivery systems, and propose conceptual frameworks to guide the development of precise, context-aware autophagy interventions. This review provides an updated and integrative perspective that bridges molecular mechanisms, host–microbiota crosstalk, and translational opportunities in autophagy-related diseases.

## 1. Introduction

Autophagy is an evolutionarily conserved lysosome-dependent degradation system that dynamically eliminates damaged organelles, misfolded proteins, and invading pathogens via lysosomal pathways, thereby maintaining cellular homeostasis and energy balance [1]. The process involves multiple sequential steps, including initiation by the ULK1 kinase complex [2], nucleation of autophagosomes mediated by the PI3KC3 complex, and membrane elongation driven by the ATG8/LC3 lipidation system [3]. Since Yoshinori Ohsumi was awarded the 2016 Nobel Prize in Physiology or Medicine for elucidating the molecular mechanisms of autophagy [4], research in this field has advanced rapidly, revealing the multifaceted roles of autophagy in cell biology and disease regulation. Autophagy has broad physiological significance, serving as an adaptive response to nutrient deprivation, oxidative stress, and other cellular stimuli. Autophagy facilitates fundamental biological processes, including embryonic development and immune surveillance. Current pharmacological interventions involve mTOR inhibition via rapamycin [5] and lysosomal modulation via chloroquine [6]. Nanomedicine delivery platforms are being developed to achieve precise regulation of autophagic pathways, particularly in the context of infectious diseases [7]. Furthermore, evidence indicates that the microbiota–gut–brain axis functions as a systemic regulator of autophagic flux [8]. This review synthesizes the molecular mechanisms of autophagy and its regulatory roles in neurological and metabolic disorders. We specifically analyze the impact of host–microbiota interactions on autophagic dynamics. The objective is to provide a theoretical framework for context-aware, targeted interventions.

## 2. Autophagy: Types and Molecular Mechanisms

### 2.1. Macroautophagy

Macroautophagy was initially described as a lysosomal transport process for stress-induced cellular homeostasis. Current research indicates it has distinct, sometimes opposing, functions in tumorigenesis and neuroprotection [9,10]. The initiation of macroautophagy involves a complex molecular cascade. Liquid–liquid phase separation (LLPS) acts as a biophysical mechanism for the rapid assembly of the ULK complex (ULK1/2/FIP200) under mTOR regulation [11]. Subsequently, the class III PI3K complex initiates nucleation. Membrane expansion and lysosomal fusion are facilitated by the ATG12–ATG5–ATG16L1 system, LC3–PE conjugation, and SNARE proteins [11]. The regulatory network of macroautophagy is multifactorial. For example, calcium signaling activates autophagy via the CaMKKbeta-AMPK pathway by inhibiting mTOR, while endoplasmic reticulum (ER)-bound Bcl-2 concurrently suppresses this process [12]. Current research often overlooks the limits of this cellular regulatory capacity. Prolonged or extreme macroautophagic stimulation in quiescent cells can exceed lysosomal degradation limits. This creates functional competition between autophagic flux and lysosomal integrity. Consequently, excessive stimulation may alter cell fate decisions and delay apoptotic clearance, complicating its therapeutic application.

### 2.2. Selective Autophagy

Selective autophagy degrades specific intracellular targets, such as damaged mitochondria (mitophagy) or pathogens. This specificity depends on interactions between ATG8-family proteins and LC3-interacting regions (LIR) on cargo receptors [13,14]. Recent studies show this mechanism is highly dynamic. Multiple substrates, including glycogen and ribosomal proteins, can attach to lipidated ATG8 via receptors (e.g., 45, Hab1) or the Snx4-20 heterodimer. This multi-substrate integration increases degradation efficiency [15,16,17]. Selective autophagy demonstrates species-specific evolutionary conservation. In plants, the VISP1 receptor targets the viral silencing suppressor CMV2b to reduce pathogenicity [18]. However, pathogens and tumor cells frequently hijack this machinery. In glioblastoma, the RNA editing enzyme ADAR1 utilizes p62-mediated selective autophagy to promote tumor growth and temozolomide (TMZ) resistance. Similarly, the KSHV virus uses the NDP52 receptor to degrade P-bodies and facilitate viral replication [19,20,21]. The regulation of selective autophagy also involves post-translational modifications, such as TBK1-dependent phosphorylation of TAX1BP1, which enhances ubiquitin binding during autophagosome assembly. These findings indicate that while selective autophagy is a potential therapeutic target, its broad pharmacological modulation may unintentionally promote viral replication or tumor progression.

### 2.3. Chaperone-Mediated Autophagy

Chaperone-mediated autophagy (CMA) operates as a highly specific, non-vesicular degradation pathway. Cytosolic proteins containing a KFERQ pentapeptide motif are recognized by the heat shock cognate protein 70 (Hsc70). The foundational mechanism of CMA was delineated with the identification of the lysosomal membrane protein 2A (LAMP2A) as the essential receptor mediating substrate unfolding and direct translocation into the lysosome [22]. Recent original investigations reveal that CMA integrates key intracellular signaling and inflammatory pathways. For instance, in microglia, CMA actively degrades the NF-κB co-activator p300, which fundamentally suppresses uncontrolled NLRP3 inflammasome activation during neuroinflammation [23]. In contrast, glioma-associated CMA dysfunction stabilizes hypoxia-inducible factor (HIF)-1 alpha, thereby inducing chemo-resistance. A major limitation in CMA-targeted oncology research is the risk of systemic toxicity; inhibiting CMA to sensitize tumors to chemotherapy may inadvertently accelerate neurodegeneration by halting the clearance of neurotoxic protein aggregates [24].

### 2.4. Microautophagy

**Figure 1:**
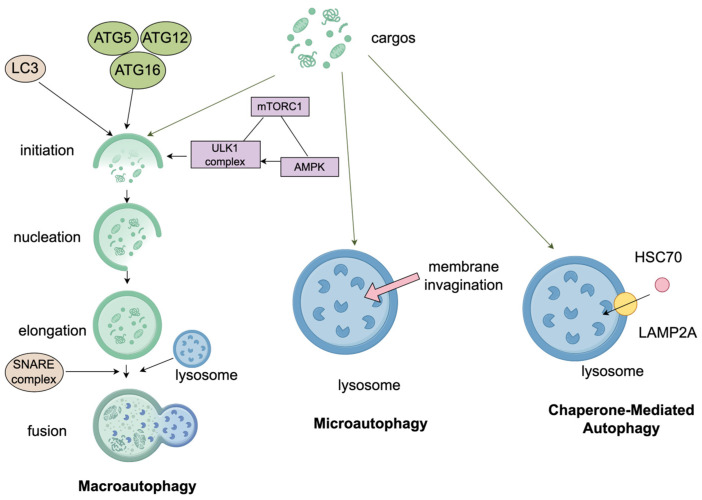
Mechanisms of the three main types of autophagy: macroautophagy, microautophagy, and chaperone-mediated autophagy (CMA). Macroautophagy involves the ULK1 initiation complex regulated by mTORC1 and AMPK, and the autophagosome formation module comprising ATG5, ATG12, ATG16, and LC3. CMA is mediated by HSC70 and LAMP2A, while lysosomal fusion is facilitated by SNARE proteins. (Create with Figdraw: https://www.figdraw.com/; accession date: 7 September 2025).

Microautophagy involves the direct invagination of the lysosomal or vacuolar membrane to engulf cytosolic cargo, exhibiting distinct morphological subtypes in processes such as micropexophagy and micromitophagy. Groundbreaking mechanistic studies in mammalian models definitively demonstrated that membrane scission in microautophagy requires highly coordinated interactions between ATG proteins and the endosomal sorting complexes required for transport (ESCRT) [25]. While microautophagy fundamentally functions to repair lysosomal membranes and maintain intracellular homeostasis, its study in human diseases faces methodological bottlenecks. The lack of specific molecular markers for mammalian microautophagy limits precise in vivo tracking and complicates distinguishing its function from macroautophagy9. Advancing clinical applications requires prioritizing the discovery of specific mammalian markers and clarifying the synergistic crosstalk between these parallel autophagic pathways (Figure 1).

## 3. Autophagy in Neurological Disorders

**Figure 2:**
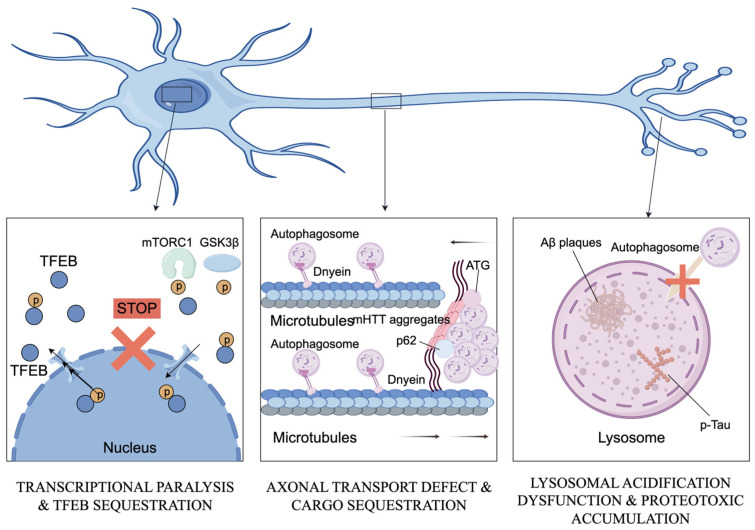
Mechanisms of neuronal dysfunction associated with autophagic impairment in neurodegenerative diseases. This figure illustrates three core pathological mechanisms: transcriptional paralysis via mTORC1/GSK3β-mediated TFEB sequestration in the cytoplasm, axonal transport defects caused by dynein dysfunction and sequestration of autophagic cargo (e.g., ATG proteins) by aggregated proteins (Aβ plaques, p-Tau, mHTT), and lysosomal acidification dysfunction leading to impaired autophagosome-lysosome fusion. These interconnected mechanisms collectively block autophagic flux and promote proteotoxic accumulation, contributing to neuronal loss in Alzheimer’s disease, Parkinson’s disease, Huntington’s disease, and amyotrophic lateral sclerosis. This model hypothesizes that the convergence of transcriptional silencing and physical cargo sequestration creates an irreversible ‘autophagic lock’ in neurodegeneration. (Create with Figdraw: https://www.figdraw.com/; accession date: 9 February 2026).

Neurodegenerative diseases, including Alzheimer’s (AD) and Parkinson (PD), Huntington’s disease (HD), and even amyotrophic lateral sclerosis (ALS), all have one fundamental weakness or characteristic: the gradual decline of them leads to progressive and irreversible loss of neuronal function. The very nature of neurons as post-mitotic cells incapable of diluting their toxic by-products via division renders them highly susceptible to the needful strict proteostasis systems to survive. Mechanistically, these different clinical entities meet in a common pathologic defect: the failure of the autophagy-lysosome pathway. The breakdown is not a mere side effect but rather a core cause of the pathogenesis and results in massive accumulation of protein aggregates, such as amyloid-β, α-synuclein, and mutant huntingtin, due to dysregulated key signaling hubs, such as mTOR and AMPK, and particular defects in CMA. Crucially, this proteotoxic stress is exacerbated by mitochondrial dysfunction; the high metabolic requirements of the brain coupled with dysfunctional mitophagy are unable to eliminate damaged organelles, leading to the initiation of an uncontrolled process of oxidative stress and neuroinflammation that overwhelms cellular resilience and eventually leads to the demise of neurons (Figure 2).

### 3.1. Transcriptional Dysregulation and Autophagy Initiation Failure

The effective maintenance of cellular proteostasis is fundamentally dependent on the coordinated activation of the Coordinated Lysosomal Expression and Regulation (CLEAR) gene network, a process largely governed by the master transcription factor TFEB. The revolutionary identification of TFEB as a central regulator [26] underscores why its nuclear exclusion is a common pathological bottleneck. In AD and PD, this failure is primarily enzymatic: TFEB is excessively phosphorylated by the mTORC1-GSK3β axis, which leads to its aberrant cytoplasmic retention and a failure to clear amyloid-β and Tau aggregates [27,28]. This enzymatic suppression is further intensified in PD by PARP1-induced poly-ADP-ribosylation, which directly obstructs TFEB’s nuclear import [29]. Complementing these enzymatic defects, ALS presents a more mechanical barrier to autophagy initiation. Mutations in C9orf72 are now known to disrupt the stoichiometric interaction between the nucleoporin POM121 and the Sigma-1 receptor [30]. This defect physically compromises the nuclear pore complex, trapping TFEB in the cytosol and facilitating the pathological spread of TDP-43 [31]. Interestingly, this transcriptional “paralysis” extends even to psychiatric contexts such as major depressive disorder, where hippocampal SIRT1 deficiency leads to the repression of essential autophagy genes like ATG5 [32]. The mechanistic link between SIRT1 and the CLEAR network was recently clarified by evidence showing that SIRT1 must directly deacetylate TFEB at the Lys116 residue to permit its nuclear entry [33].

While these models offer a consistent mechanistic narrative, their clinical translatability warrants critical caution. A significant methodological limitation is that much of this data stems from acute, high-dose protein overexpression in transgenic mice, which may artificially amplify the collapse of the CLEAR network beyond what is observed in the chronic, decades-long progression of human disease. Furthermore, the temporal dynamics of TFEB activation are still debated; some clinical evidence suggests an initial compensatory upregulation of autophagy in the prodromal stages of AD, implying that TFEB exhaustion may be a consequence of late-stage systemic failure rather than an initiating driver [34]. Resolving these biological nuances is essential for developing spatiotemporal-specific modulators that avoid the risks of lysosomal over-activation.

### 3.2. Impaired Cargo Recognition and Autophagic Transport Deficits

Beyond the failure to initiate autophagy, the physical presence of protein aggregates actively sabotages the machinery, causing autophagic flux impairment or vesicular transport stasis. In HD, for instance, mature fibrillar mutant huntingtin aggregates act as competitive decoys that sequester selective receptors like p62 and essential ATG proteins [35]. This sequestration effectively depletes the functional pool of these components, arresting autophagosome maturation in distal neurites [36]. A parallel mechanism of physical obstruction exists in the non-vesicular CMA pathway during PD pathogenesis, where α-Synuclein binds to the LAMP2A receptor with abnormally high affinity, physically clogging the translocation channel [8]. These transport deficits are fundamentally compounded by downstream degradation failures, particularly in AD. Original biochemical investigations have pinpointed the primary bottleneck to a failure in lysosomal acidification, driven by defects in the v-ATPase assembly [37]. This failure in the terminal digestive step leads to a massive accumulation of undigested autophagic vacuoles (AVs), a pathology exacerbated by mutations in LC3-associated endocytosis [38].

The “aggregate-blockade” hypothesis, while widely accepted, requires a critical re-evaluation of its methodological underpinnings. For instance, much of the evidence for LAMP2A clogging remains limited to in vitro isolated lysosome assays, and quantifying this obstruction within intact, living neural networks remains a significant technical hurdle. Moreover, the role of cargo accumulation is increasingly contested by emerging perspectives on liquid–liquid phase separation (LLPS), which suggest that p62-mHTT assemblies might actually be protective scaffolds designed to isolate toxic oligomers rather than mere “garbage” causing congestion [39]. Such conflicting interpretations highlight the unresolved bottleneck of substrate-state dependence: while existing activators can clear early-stage non-fibrillar oligomers, they may paradoxically worsen the lethal accumulation of AVs when applied to late-stage pathology characterized by dense, insoluble plaques.

### 3.3. Defective Mitophagy and the Neuroinflammation Cycle

Failure of the autophagy-lysosome pathway to clear damaged organelles, notably mitochondria, creates a vital pathophysiological link between proteostasis collapse and chronic neuroinflammation. In healthy cells, the PINK1-Parkin pathway marks dysfunctional mitochondria and facilitates their autophagic engulfment by recruiting LC3/GABARAP [40]. This targeted mitophagy is severely impaired in PD, frequently due to LRRK2 mutations. These mutations phosphorylate Rab proteins, which in turn blocks the recruitment of essential mitophagy receptors [41]. When defective mitochondria escape degradation, they leak excessive reactive oxygen species (ROS) and mitochondrial DNA (mtDNA) into the cytosol, acting as major neuroinflammatory triggers. Evidence from major depressive disorder (MDD) models shows that ROS accumulation hyperactivates the NLRP3 inflammasome in microglia [42]. Consequently, these activated microglia release pro-inflammatory cytokines like IL-1β. This triggers NF-κB signaling, which further inhibits autophagic flux. The result is a destructive feedback loop where mitochondrial decline and escalating inflammation reinforce each other.

Although this loop is evident in vitro, translating these findings to human pathology requires careful assessment. Current research relies heavily on acute in vivo stimulation, such as high-dose lipopolysaccharide (LPS) injections. Such models rarely reflect the chronic, low-grade “inflammaging” seen in the aging human brain. Additionally, the causal order of autophagic decline and neuroinflammation remains debated. While most theories label autophagic failure as the primary cause, longitudinal data suggests that early, brief inflammatory bursts might actually trigger compensatory autophagy as a defense. Pathway exhaustion may only occur after long-term exposure. Addressing this causal uncertainty is essential for creating treatments that decouple mitophagy defects from inflammasome activation without causing broad immunosuppression.

### 3.4. The Microbiota–Gut–Brain Axis: Systemic Regulation

Beyond cell-autonomous defects, the microbiota–gut–brain axis (MGBA) functions as a key systemic regulator of neuronal proteostasis. Initial evidence for this connection came from studies demonstrating that gut microbiota are essential for the motor deficits and neuroinflammation seen in α-Synuclein overexpressing mice [43]. Under normal conditions, symbiotic gut bacteria generate metabolites like short-chain fatty acids (SCFAs). These SCFAs cross the blood–brain barrier and stimulate neuronal autophagic flux through the AMPK-mTOR axis [44]. In contrast, gut dysbiosis impairs the intestinal epithelial barrier. This “leaky gut” state permits bacterial endotoxins, such as LPS, to enter systemic circulation. Once circulating, these toxins activate TLR4 signaling to trigger neuroinflammation, which subsequently inhibits neuronal autophagy. This process accelerates the buildup of Aβand α-Synuclein. These mechanisms support the “gut-first” hypothesis in PD. This theory suggests that pathogenic aggregation starts in the enteric nervous system and moves to the brain through the vagus nerve.

**Figure 3:**
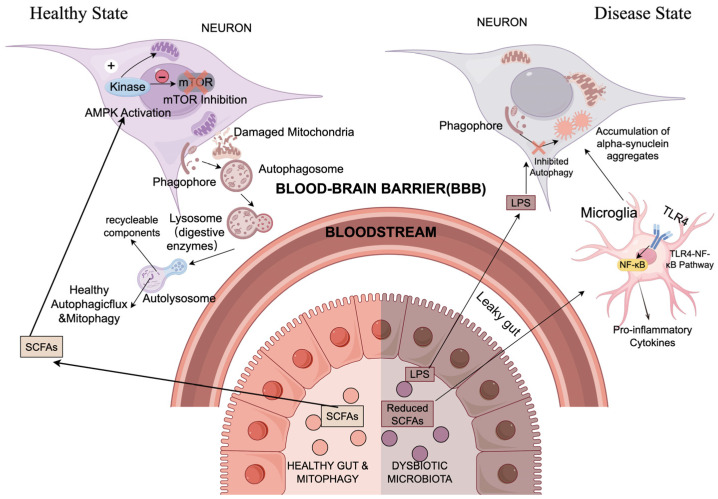
Contrasting mechanisms of the microbiota–gut–brain axis (MGBA) in regulating neuronal autophagy under healthy and dysbiotic conditions. Symbols: "+" = pathway activation; "-" = autophagy inhibition. In the healthy state, balanced gut microbiota produce short-chain fatty acids (SCFAs, e.g., butyrate) that cross the blood–brain barrier (BBB), activate the AMPK/mTOR pathway, and enhance autophagic flux (including mitophagy) to clear damaged mitochondria and misfolded proteins. In the dysbiotic state, reduced SCFA production and increased lipopolysaccharide (LPS) release disrupt the intestinal barrier; LPS enters the bloodstream, crosses the BBB, and triggers neuroinflammation via the TLR4-NF-κB pathway, which inhibits neuronal autophagy and promotes α-synuclein aggregation. Illustrating the hypothesis that gut-derived metabolites act as systemic switches for neuronal autophagic flux. (Create with Figdraw: https://www.figdraw.com/; accession date: 9 February 2026).

Despite the novel systems-biology perspective offered by the MGBA, clinical translation faces significant hurdles. Most human studies establish merely a correlation, rather than causation, between gut dysbiosis and neurodegeneration. While fecal microbiota transplantation (FMT) has reactivated autophagy in rodent models [45], large-scale clinical validation in humans is still lacking. Furthermore, profound interspecies differences in microbiome composition mean that results from highly controlled laboratory settings often fail to replicate in heterogeneous human populations. A key scientific challenge remains identifying specific microbial metabolites—such as certain SCFAs or mitophagy-inducers like Urolithin A—that can selectively modulate brain autophagy without causing systemic disturbances [46]. Whether the “gut-first” hypothesis applies universally or only to specific clinical subtypes remains to be validated by prospective longitudinal studies (Figure 3).

### 3.5. Convergent Autophagy-Targeted Therapeutic Strategies

**Figure 4:**
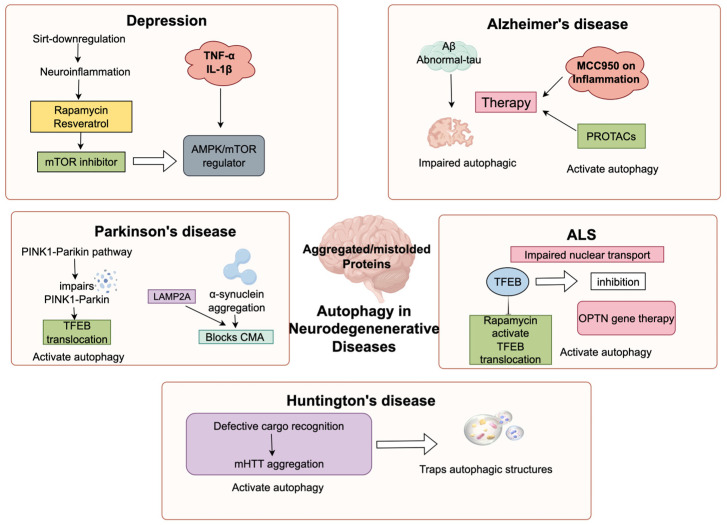
Mechanisms and therapeutic strategies of autophagy in five neurodegenerative diseases: depression, Alzheimer’s disease (AD), Parkinson’s disease (PD), amyotrophic lateral sclerosis (ALS), and Huntington’s disease (HD). In depression, downregulation of sirtuins triggers neuroinflammation, and TNF-α and IL-1β regulate autophagy via the AMPK/mTOR pathway; interventions such as rapamycin can modulate this pathway. In AD, autophagy is impaired due to Aβ accumulation and abnormal Tau protein, and autophagy can be activated by MCC950 via AMPK/mTOR or PROTACs-based approaches. In PD, dysregulation of the PINK1–Parkin pathway and α-synuclein aggregation inhibit relevant molecular processes, whereas TFEB translocation activates autophagy. In ALS, nuclear transport defects occur, and autophagy can be induced by rapamycin-mediated TFEB activation or Sox2–OPTN gene therapy. In HD, defects in cargo recognition lead to mHTT aggregation and sequestration of autophagic structures; autophagy activation can alleviate pathology. Overall, these examples highlight the association between autophagy dysregulation and neurodegenerative diseases and illustrate the therapeutic potential of targeting autophagy. (Create with Figdraw: https://www.figdraw.com/; accession date: 30 January 2026).

Given the profound mechanistic convergence in the collapse of the autophagy-lysosome pathway across diverse neurological disorders, contemporary therapeutic innovation is shifting from strictly disease-specific symptomatic treatments toward unified strategies centered on restoring neuronal autophagic flux. The most classical approach in this domain involves broad-spectrum mTOR inhibitors, such as rapamycin. By disinhibiting autophagy, these agents have demonstrated significant potential in promoting the clearance of toxic aggregates in preclinical models of AD, PD, and HD [5,47]. Beyond general modulators, current research is shifting toward high-precision interventions. In Alzheimer’s disease (AD) studies, for example, researchers are developing proteolysis-targeting chimera (PROTAC) molecules. These are engineered to identify and eliminate Tau aggregates through the autophagic system [48]. Progress in Amyotrophic Lateral Sclerosis (ALS) treatments has likewise moved toward genetic and receptor-level repair. One approach uses SIGMAR1 agonists, such as pridopidine, to restore the TFEB-mediated transcriptional network [30]. Another strategy involves AAV9-based gene therapy to replace missing optineurin (OPTN), which restarts the specific mitochondrial clearance process [49]. Additionally, evidence shows that standard antidepressants can trigger autophagy via protein kinase A-dependent pathways. This discovery suggests a new method for breaking the neuroinflammatory feedback loops associated with Major Depressive Disorder (MDD) [42,50] (Figure 4).

The clinical transition of these promising therapies, however, is not without its perils. Despite the success of autophagy activators in transgenic models, their human application is obstructed by formidable pharmacokinetic and safety hurdles. Primarily, blood–brain barrier (BBB) permeability remains a decisive bottleneck; many potent modulators, particularly large macromolecular degraders and viral vectors, fail to achieve therapeutically effective concentrations within the central nervous system upon systemic administration. Secondly, the fundamental nature of autophagy as a universal homeostatic mechanism poses a risk of systemic toxicity. Blindly upregulating autophagic flux across all tissues may inadvertently disrupt the metabolism of healthy peripheral organs or even trigger autophagic cell death upon hyperactivation. Perhaps the most critical obstacle is the current lack of reliable, non-invasive in vivo biomarkers. Without the ability to dynamically monitor autophagic flux within the living human brain, it remains exceptionally difficult to ascertain target engagement or to define a safe “optimal therapeutic window.” Addressing these translational gaps—through advanced nanodelivery systems and the identification of cerebrospinal fluid or blood exosome biomarkers—represents the next essential frontier for autophagy-targeted medicine.

## 4. Autophagy in Metabolic Diseases

Metabolic disorders, including obesity, type 2 diabetes (T2D), and non-alcoholic fatty liver disease (NAFLD), represent a state of energy homeostasis failure. These conditions are characterized by insulin resistance, lipotoxicity, and chronic inflammation [51]. Autophagy functions as a core regulator of cellular quality control and metabolic plasticity; its malfunction is a primary cause of metabolic dysregulation [52]. Autophagic failure in adipose tissue during obesity drives maladaptive inflammatory responses, while the breakdown of β-cell autophagy in T2D results in organelle malfunction and impaired insulin response [53,54]. Current research indicates that these disorders share common autophagic failure patterns, primarily converging on nutrient-sensing dysregulation and organelle-mediated inflammation.

### 4.1. Convergence on the AMPK/mTOR Signaling Axi

Obesity is a systemic metabolic disease that significantly increases the risk of T2D, metabolic-associated fatty liver illness, and cardiovascular diseases [55]. A shared mechanism across these metabolic states is the disruption of the nutrient-sensing AMPK/mTOR axis. Under physiological conditions, nutrient starvation recruits AMPK to initiate autophagy via ULK1 [56]. Conversely, chronic nutrient excess hyperactivates mTORC1 and suppresses AMPK signaling [56]. This signaling imbalance leads to lipotoxicity and inflammation, exhibiting distinct organ-specific bottlenecks [57]. The downstream consequences share a common autophagic impairment. In adipose tissue, an increase in CD36 transporters causes lysosomal calcium overload. This process stops the breakdown of lipid droplets [58]. Within the liver, the primary lysosomal regulator, TFEB, fails to move into the nucleus, which prevents lysosomal genesis [58]. Similarly, in Type 1 Diabetes (T1D), faulty autophagosome-lysosome connections lead to p62 accumulation. This lack of LC3-LAMP1 colocalization triggers β-cell apoptosis [59]. The AMPK/mTOR axis serves as a general framework, yet local microenvironments significantly shift these pathways. For example, metabolites like lactate can trigger autophagy through the ERK/p90RSK pathway, bypassing mTOR. Additionally, TGF-β/Smad signaling mediators help determine β-cell survival [60]. Such metabolic diversity indicates that using broad-spectrum autophagic modulators might result in inconsistent clinical outcomes.

### 4.2. Autophagy-Inflammasome Crosstalk and Organelle Quality Control

The crosstalk between autophagy and the NLRP3 inflammasome is a critical mechanistic hub in metabolic diseases. Autophagy functions to clear damaged mitochondria that produce reactive oxygen species [61]; this process fails during diabetic complications, leading to NLRP3 hyperactivation [62]. In T2D, selective mitophagy is initially activated to maintain homeostasis [63]. However, chronic metabolic stress eventually overwhelms this adaptation, leading to damaged organelles and islet disintegration [59]. The causal relationship between autophagic decline and metabolic inflammation requires strict evaluation. Recent evidence indicates that natural substances like kaempferol normalize metabolic imbalance in diet-induced obese (DIO) mice by targeting the mitochondrial protein TUFM to revive autophagy [64]. Yet, the evolutionary conservation of these pathways, such as the Stb5 transcription factor controlling autophagy through NADPH synthesis and redox status in the yeast Saccharomyces cerevisiae [65], does not fully recapitulate the multi-organ complexity of human metabolic syndromes.

### 4.3. Implications for Therapeutic Interventions

**Figure 5:**
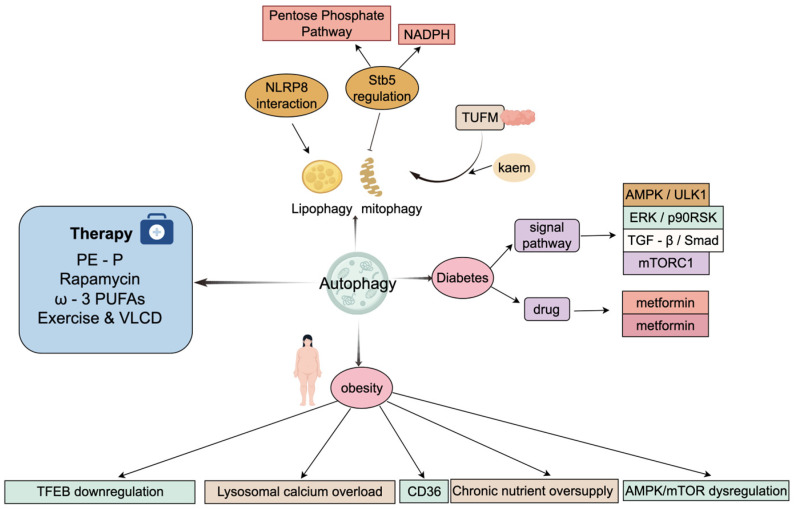
Autophagy in metabolic diseases (obesity and diabetes) and potential intervention strategies. Autophagy participates in lipid engulfment, mitophagy, and related processes, and is regulated by mechanisms including the pentose phosphate pathway, NLRP8 interactions, and Stb5. In obesity, autophagy is impaired via multiple pathways, including TFEB downregulation, lysosomal calcium overload, CD36-mediated lipid uptake, chronic nutrient excess, and AMPK/mTOR imbalance. In diabetes, autophagy can be modulated through AMPK/ULK1 signaling or pharmacological agents such as metformin. Additionally, interventions including PE-P, rapamycin, ω-3 polyunsaturated fatty acids, exercise, and very-low-calorie diets can improve obesity and diabetes by regulating autophagic activity. (Create with Figdraw: https://www.figdraw.com/; accession date: 30 January 2026).

Therapies capable of restoring this fragile signaling state are gaining emphasis [66]. Pharmacologic interventions, including Metformin and GLP-1 receptor agonists, reconnect the AMPK axis to shield cells against glucotoxicity. Concurrently, non-pharmacologic interventions like exercise and Vitamin D3 supplementation aim to resolve dysregulated autophagic flux [67], providing an opportunity to protect functional β-cell mass and stop disease onset [68]. Furthermore, emerging treatments such as marine-derived plasmalogens (PE-P), rapamycin, and ω-3 polyunsaturated fatty acids utilize various pathways (e.g., PPAR-γ) to increase autophagy, ultimately diminishing systemic lipotoxicity and metabolic hazard [69,70,71] (Figure 5). The fundamental challenge in metabolic autophagy therapeutics is achieving tissue-specific targeting. Since autophagy serves critical homeostatic functions, systemic pharmacological activation risks unintended consequences, necessitating the development of precise delivery systems. To synthesize the diverse pharmacological landscape discussed above, Table 1 summarizes the key autophagy-targeted modulators, their distinct mechanisms of action, and their current clinical stages across different pathological contexts.

## 5. Future Research Directions and Challenges

Despite significant progress in understanding the molecular mechanisms of autophagy, translating these discoveries into clinical therapies faces several critical challenges. First, there is a fundamental lack of reliable, non-invasive biomarkers to monitor autophagic flux in vivo. Current clinical studies emphasize the diagnostic potential of measuring autophagy-related proteins in patient biofluids. For example, measuring p62 levels and endo-lysosomal markers in cerebrospinal fluid (CSF) offers a way to monitor real-time autophagic changes in the brain [75,76]. Research on extracellular vesicles also shows that exosomes can indicate impaired autophagic clearance. This makes them a highly accessible platform for biomarkers [77]. A second challenge involves pharmacokinetic barriers. Specifically, the blood–brain barrier (BBB) strictly limits how well macromolecular modulators reach the central nervous system [78]. To solve this, new therapies use advanced delivery engineering. Nanomedicine, including targeted nanovesicles and multifaceted nanoparticles, has improved drug penetration across the BBB in neurodegenerative models [79,80]. Furthermore, researchers are testing physical methods like focused ultrasound. This technique temporarily increases BBB permeability to help lysosomal therapies enter the brain [81].

Finally, the context-dependent nature of autophagy poses severe safety concerns. As extensively reviewed in the recent literature, autophagy exerts context-dependent or opposing effects; it suppresses early tumorigenesis but actively promotes chemoresistance in advanced stages [82]. Consequently, broad-spectrum modulators frequently trigger unintended off-target toxicity and systemic metabolic disruptions [72]. Balancing these complex effects requires stringent spatiotemporal control, a major toxicological challenge that future nanotherapeutics must address to avoid exacerbating cellular stress [83]. Integrating targeted nanotechnology with a systems-biology approach will be crucial to unlocking the full clinical potential of precision autophagy medicine.

## 6. Conclusions

Synthesizing the diverse molecular environment discussed in this section, it becomes clear that autophagy cannot be considered as one single pathway, but rather as a high-level integrated network. The network directs cellular fate with a precision hierarchy: mass degradation by coordinated action of ULK-PI3KC3 complexes and Rab-dependent vesicular transport on one side, and the exquisite substrate selectivity of CMA, and receptor-based selective autophagic pathways such as mitophagy and lipophagy on the other. Although this constant regulatory adaptation allows cells to adapt dynamically to metabolic changes and stresses, the translation of these mechanical cues into clinical practice unveils a complex situation of a Janus-faced reality. Autophagy is a central dichotomy in disease treatment. Restoration interventions, e.g., the administration of rapamycin to improve neurodegeneration, PROTAC-directed degradation of Tau aggregates in Alzheimer’s disease, or marine-derived plasmalogen use to maintain neuronal proteostasis, demonstrate that enhancing autophagic flux is essential for homeostasis. Nevertheless, significant paradoxes and limitations exist for clinical implementation. A key challenge lies in the context-dependent activity of autophagy, which requires precise temporal and spatial control to balance protective and detrimental effects. Additionally, existing modulators are frequently blunt instruments lacking tissue-specificity and pose risks of systemic toxicity, while specific pathways of mammalian microautophagy and systemic interactions between macroautophagy and CMA remain largely unexplored. Therefore, the future of autophagy-based therapy must shift toward precise intervention. To achieve this, spatiotemporally sensitive devices that target genetic backgrounds will need development, nanotechnology for targeted delivery, and a systems-biology framework to decode synergistic regulation between autophagic subtypes. Finally, by bridging fundamental molecular mechanisms with clinical complexity, targeted autophagy modulation holds transformative potential for treating neurodegenerative diseases.

## Figures and Tables

**Table 1:** Summary of key autophagy-targeted modulators, their mechanisms of action, and clinical development stages across neurological and metabolic disorders.

Class	Agent	Mechanism of Action	Target Disease/Indication	Clinical Stage	References
mTORC1 inhibitor	Rapamycin (Sirolimus)	Releases the molecular disinhibiting on autophagy; promotes clearance of toxic aggregates and corrects anabolic locks.	Neurodegeneration (AD, PD, HD), Diet-induced Obesity.	Phase 2	[5,47]
Lysosomal function modulator	Chloroquine/HCQ	Inhibits autophagy late-stage flux; disrupts stress-responsive survival capabilities.	Advanced Oncology.	Phase 1/2	[6]
PI3KC3 inhibitor	SAR405	Inhibits autophagy initiation (often used in combination therapies).	Advanced Oncology.	Preclinical	[72]
Targeted degrader	PROTACs (Small-molecule)	Selectively recognizes and degrades Tau aggregates via the autophagic machinery.	Alzheimer’s Disease.	Phase 1/Preclinical	[48]
SIGMAR1 agonist	Pridopidine	Rescues the TFEB-mediated transcriptional network to restore autophagy initiation.	Amyotrophic Lateral Sclerosis (ALS).	Phase 3	[30,73]
Gene therapy	AAV9-OPTN	Replaces lost optineurin (OPTN) to reactivate the targeted mitochondrial clearance network.	Amyotrophic Lateral Sclerosis (ALS).	Preclinical	[49]
AMPK axis activator	Metformin	Reconnects the AMPK axis to shield cells against glucotoxicity.	Type 2 Diabetes (T2D).	Approved	[74]
GLP-1 receptor agonist	GLP-1 analogues (e.g., Semaglutide)	Reconnects the AMPK axis to resolve dysregulated autophagic flux.	Type 2 Diabetes (T2D), Obesity.	Approved	[74]
Protein Kinase A modulator	Classical antidepressants	Induce autophagy through protein kinase A-dependent mechanisms to disrupt neuroinflammation.	Major Depressive Disorder (MDD).	Approved	[42,50]
Mitochondrial targeter	Kaempferol	Targets the mitochondrial protein TUFM to revive autophagy and normalize metabolic imbalance.	Dietary-induced Obesity (DIO).	Preclinical	[64]
Lipid modulator	Marine-derived plasmalogens (PE-P)	Promotes hypothalamic autophagy-lysosome association.	Obesity, Hepatic Steatosis.	Preclinical	[69,70,71]
PPAR-γ agonist	ω-3 polyunsaturated fatty acids	Uses the PPAR-γ pathway to increase the degree of autophagy, diminishing systemic lipotoxicity.	Obesity.	Approved/Phase 4	[69,70,71]
Mitophagy inducer	Urolithin A	Microbial metabolite that selectively modulates brain mitophagy via the Microbiota–Gut–Brain Axis.	Neurodegenerative Diseases, Aging.	Phase 1/2	[46]

## Data Availability

No new data were created or analysed in this study. Data sharing is not applicable to this article.

## Abbreviations

The following abbreviations are used in this manuscript:

AD	Alzheimer’s Disease
ATG	Autophagy-Related Gene
CMA	Chaperone-Mediated Autophagy
T1D	Type 1 Diabetes
T2D	Type 2 Diabetes
Hsc70	Heat Shock Cognate Protein 70
PD	Parkinson’s Disease
PI3KC3	Class III Phosphoinositide 3-Kinase
WTHTT	Wild-Type Huntingtin
Hab1	Habituation Protein 1
